# Identification and functional analysis of a novel de novo missense mutation located in the initiation codon of *LAMP2* associated with early onset female Danon disease

**DOI:** 10.1002/mgg3.2216

**Published:** 2023-06-08

**Authors:** Yongxiang Wang, Ming Bai, Piyi Zhang, Yu Peng, Zixian Chen, Zhiyu He, Jin Xu, Youqi Zhu, Dongdong Yan, Runqing Wang, Zheng Zhang

**Affiliations:** ^1^ Heart Center The First Hospital of Lanzhou University Lanzhou Gansu China; ^2^ Gansu Key Laboratory of Cardiovascular Diseases The First Hospital of Lanzhou University Lanzhou Gansu China; ^3^ Gansu Provincial Clinical Research Center for Cardiovascular Diseases The First Hospital of Lanzhou University Lanzhou Gansu China; ^4^ The First Clinical Medical School, Lanzhou University Lanzhou Gansu China; ^5^ Department of Radiology The First Hospital of Lanzhou University Lanzhou Gansu China

**Keywords:** autophagy, Danon disease, haploinsufficiency, initiation codon mutation, lysosome‐associated membrane glycoprotein 2, X chromosome inactivation

## Abstract

**Background:**

Danon disease is characterized by the failure of lysosomal biogenesis, maturation, and function due to a deficiency of lysosomal membrane structural protein (*LAMP2*).

**Methods:**

The current report describes a female patient with a sudden syncope and hypertrophic cardiomyopathy phenotype. We identified the pathogenic mutations in patients by whole‐exon sequencing, followed by a series of molecular biology and genetic approaches to identify and functional analysis of the mutations.

**Results:**

Suggestive findings by cardiac magnetic resonance (CMR), electrocardiogram (ECG), and laboratory examination suggested Danon disease which was confirmed by genetic testing. The patient carried a novel de novo mutation, *LAMP2* c.2T>C located at the initiation codon. The quantitative polymerase chain reaction (qPCR) and Western blot (WB) analysis of peripheral blood leukocytes from the patients revealed evidence of *LAMP2* haploinsufficiency. Labeling of the new initiation codon predicted by the software with green fluorescent protein followed by fluorescence microscopy and Western blotting showed that the first ATG downstream from the original initiation codon became the new translational initiation codon. The three‐dimensional structure of the mutated protein predicted by alphafold2 revealed that it consisted of only six amino acids and failed to form a functional polypeptide or protein. Overexpression of the mutated *LAMP2* c.2T>C showed a loss of function of the protein, as assessed by the dual‐fluorescence autophagy indicator system. The mutation was confirmed to be null, AR experiments and sequencing results confirmed that 28% of the mutant X chromosome remained active.

**Conclusion:**

We propose possible mechanisms of mutations associated with haploinsufficiency of *LAMP2*: (1) The inactivation X chromosome carrying the mutation was not significantly skewed. However, it decreased in the mRNA level and the expression ratio of the mutant transcripts; (2) The identified mutation is null, and the active mutant transcript fails to translate into the normal *LAMP2* proteins. The presence of haploinsufficiency in *LAMP2* and the X chromosome inactivation pattern were crucial factors contributing to the early onset of Danon disease in this female patient.

## Graphical abstract

We report a novel initiation codon mutation associated with Danon disease in young women. Through identification and functional analysis, our results indicate that the mutation is null and that the X chromosome inactive state affects the expression of the mutant transcript, which is responsible for early‐onset cardiomyopathy in this patient.

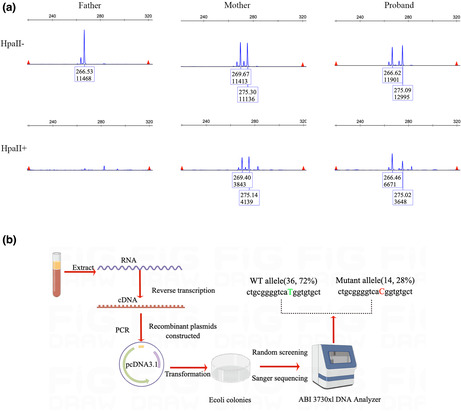

## 1 INTRODUCTION

Danon disease (DD) (OMIM: No. 300257) is a multisystem disorder of the skeletal muscle, myocardium, and retina, leading to some patients with intellectual and cognitive dysfunction. It is a rare X‐linked autophagic vacuolar myopathy characterized by high penetrance and severe cardiomyopathy (Brambatti et al., 2019). Usually, male hemizygotes show more severe clinical phenotypes than female heterozygotes. The disorder develops earlier in men who more frequently require heart transplants than women (Taylor & Adler, 2020). Disease onset often first appears in the heart, with gender differences being reflected in the severity since 70.3% of women present with hypertrophic cardiomyopathy (HCM) and 29.3% with dilated cardiomyopathy (DCM) while 96.2% of men have HCM (Brambatti et al., 2019). Despite the milder phenotype among female patients, women's cardiac lesions may present as an isolated clinical feature as severe as those in men (Cenacchi et al., 2020). Wolff–Parkinson–White (WPW) rhythms were present in 27%–35% of cases from the early stages (Boucek et al., 2011; Dougu et al., 2009; Lopez‐Sainz et al., 2019), along with atrial fibrillation (63% of patients), flutter and malignant VA which may cause syncope and sudden death (Lopez‐Sainz et al., 2019; Toib et al., 2010). Echocardiography tends to show concentric left ventricular hypertrophy with a common HCM phenotype in young women (Cheng et al., 2012; Tada et al., 2010). Many female patients show an increase in serum troponin‐T and creatine kinase during acute cardiac illness (Hedberg Oldfors et al., 2015).

Formal clinical diagnostic criteria for Danon disease have not been established. Taylor & Adler (2020) defined that the diagnosis of Danon disease is usually established in a female proband with cardiac preexcitation and cardiomyopathy (either hypertrophic or dilated) by identification of a heterozygous pathogenic mutation in *LAMP2* on molecular genetic testing. For genetic testing, sequence analysis is the preferred method by detecting 95% of the pathogenic variants. Biopsy and tissue staining demonstrating the absence of the *LAMP2* protein also confirm the diagnosis, but this assay is not widely available on a clinical basis (Taylor & Adler, 2020).

*LAMP2* (Lysosome‐associated membrane glycoprotein 2) mutations located on chromosome Xq24 are known to be the major causative gene for DD (Nishino et al., 2000). *LAMP2* is critical for autophagy and mediates the fusion of the autophagosome with the lysosome to generate the autolysosome, autophagic vacuole maturation, and chaperon‐mediated protein transport to lysosomes (Hashida et al., 2015). Autophagy is a process by which cells degrade and recycle surplus or damaged intracellular components, such as lipids, glycogen, and mitochondria (Farah et al., 2020). Debris is packaged by the autophagosome, and the autolysosome degrades the cargo into small molecules for reuse. *LAMP2* mutations block autolysosome synthesis, thus resulting in the formation of numerous autophagic vacuoles containing glycogen and lipid droplets that cannot be degraded and which accumulate in DD. Other disorders in which glycogen accumulates, such as Pompe disease or Forbes disease, result from mutations in genes for the key enzymes of glucose metabolism, including *GAA*: acid α‐glucosidase and glycogen debranching enzyme. Myocardial biopsy is an alternative diagnostic method but is less acceptable than noninvasive genetic diagnosis and has demanding technical requirements. Myopathy is rarely observed in females (Boucek et al., 2011; Lopez‐Sainz et al., 2019; Sugie et al., 2002), rendering skeletal muscle biopsy unsuitable and, when present, muscle weakness is usually mild. Females with DD have been reported to be 30% weaker than healthy females (Stevens‐Lapsley et al., 2010). Milder skeletal muscle effects in females may result from later disease onset than in males which means that myocardial defects predominate (Hashida et al., 2015).

The *LAMP2* gene consists of nine exons. Exon 9 undergoes alternative splicing to produce 3 isoforms, *LAMP2*‐2a, *LAMP2*‐2b, and *LAMP2*‐2c, which share the same luminal domain but have different transmembrane and cytoplasmic domains (Rowland et al., 2016). Most *LAMP2* mutations are predicted to result in deficiency of all 3 *LAMP2*‐2 isoforms and isoform‐specific mutations have only been recorded for *LAMP2*‐2b (Clinvar database), suggesting that *LAMP2*‐2b deficiency alone is sufficient to cause the disease.

The Clinvar database contains 3 *LAMP2* variants in the initiation codon (c.1A>T, c.1A>C, c.3G>A). Of which, c.1A>T and c.3G>A were likely pathogenic or pathogenic (P/LP) but lacking functional evidence. The current study reports the case of a female patient with DD who was genetically diagnosed along with cardiac magnetic resonance imaging (CMR) findings and clinical presentation. A functional validation of the product of this previously unreported de novo mutation is presented and confirms the mechanism by which the mutation causes the disease.

## 2 MATERIALS AND METHODS

### 2.1 Ethics statement

Studies involving human participants were reviewed and approved by The First Hospital of Lanzhou University (No. LDYYLL‐2022‐405). Patients/participants provided their written informed consent to participate in this study. The study met the requirements of the Declaration of Helsinki.

### 2.2 Clinical examinations

A 20‐year‐old female patient was referred to our hospital with a 2‐year history of hypertrophic cardiomyopathy (HCM) and one occurrence of syncope. Two years ago, the patient was found to have a cardiac abnormality during an electrocardiograph (ECG) examination with sinus bradycardia and an irregular indoor conduction block. She denied having symptoms of palpitation, chest tightness, or shortness of breath. Echocardiography and CMR suggested HCM with a local narrow heart cavity. The patient was given appropriate HCM medication (details not available) but discontinued this 1 month later without the regular follow‐up. Her parents recalled that approximately 25 days before admission, the patient had sudden syncope with loss of consciousness while walking of 1‐min duration but no symptoms of incontinence, limb twitching, nausea, or vomiting were apparent. The parents recalled the ECG and head CT (Computed Tomography) taken at the time as being normal (results unavailable). The patient was admitted to our hospital and received a comprehensive examination, including somatoscopy (including muscle strength assessment by MRC grade and retinopathy by ophthalmoscope), evaluation of intellectual development by Wechsler Intelligence Scale, *physical examination* (including Holter, electrocardiogram, echocardiography, cardiac MRI), and laboratory tests (including myocardial enzymes and biochemical indicators).

### 2.3 Cell culture

Human embryonic kidney cell line, HEK293 cells (Procell: no. CL‐0005) were cultured in DMEM (Biological Industries) supplemented with 10% heat‐inactivated low‐endotoxin fetal bovine serum (Biological Industries) and penicillin–streptomycin solution (Biological Industries) at 37°C and 5% CO_2_.

### 2.4 Isolation of peripheral blood mononuclear cells from peripheral venous blood

Venous blood was drawn from the patient's elbow with the patient's informed consent into a collecting vessel containing EDTA (Ethylenediaminetetraacetic acid). PBMCs were isolated by Ficoll Paque lymphocyte isolate (GE: no. 17‐5542‐02). Briefly, 2 mL PBS was added to 2 mL anticoagulant‐treated blood (final volume: 4 mL) and transferred to a 15 mL centrifuge tube with mixing by inversion. Three milliliters of Ficoll‐paque PREMIUM was added and the tube was centrifuged at 400 ***g*** for 30 min at 18°C. The pellet containing mononuclear cells was transferred to a sterile centrifuge tube by sterile pipette, washed with PBS 400 ***g*** for 10 min at 18°C and stored in serum‐free medium at −80°C.

### 2.5 Whole exon sequencing

DNA was extracted from peripheral blood by the blood genome extraction kit (Tian Gen; no. DP304), according to the manufacturer's instructions. Whole exon and exon–intron boundaries for 20,000 genes in the human genome were captured with IDT the xGen Exsome research panel v2.0 kit. Next‐generation sequencing was performed on Illumina HiSeq 2000 sequencer at a high‐quality sequencing depth (30×). Raw data from sequencing were put under quality control by FASTQC. Clean reads were aligned to the human genome database (NCBI build 37, hg 19) using the BWA program. Insertion–deletions and single nucleotide polymorphisms (SNPs) processed by Sequence Alignment/Map tools (SAM tools) and detected by Genome Analysis Toolkit (GATK). ANNOVAR with RefSeq (hg19, from UCSC) and UCSC annotation (http://www.ncbi.nlm.nih.gov/refseq/) were applied to annotate the variants (including locations and effects on protein coding).

In the screening of pathogenic genes and pathogenic mutations, we prioritized 15 genes (*MYH7*, *MYBPC3*, *TNNT2*, *TNNI3*, *TPM1*, *MYL2*, *MYL3*, *ACTC1*, *PLN*, *FLNC*, *GLA*, *LAMP2*, *PRKAG2*, *TTR*, and *GAA*) associated with the hypertrophic cardiomyopathy phenotype were the top priority (Section of Precision Cardiovascular Medicine of Chinese Society of Cardiology et al., 2019). We defined our set of candidate variants based on allele frequency and function. Allele frequency was estimated from dbSNP (https://www.ncbi.nlm.nih.gov/snp/), 1000 Genome (http://www.ncbi.nlm.nih.gov/Ftp/), ExAC (https://exac.broadinstitute.org/), and filtered with a 0.1% cutoff value to remove possible common variants. For the functional filter, loss of function (LOF) mutations (including nonsense, splicing, and frameshift InDels) were directly harmful. Missense mutations are considered deleterious only when the prediction of in silico pathogenicity by Polyphen2 and SIFT. Finally, the variants were matched to the patient's clinical characteristics through the OMIM database. The resulting mutation sites were graded by ACMG guidelines.

### 2.6 Plasmid construction

Plasmids of pcDNA3.1^+^ with WT and Mut *LAMP2*‐cDNA (NM_013995.2) were acquired from HITRO Bio‐Tech company. They were used to verify the effect of the mutations on the *LAMP2* protein expression levels. In addition, Plasmids of PEGFP‐N1 with WT and Mut *LAMP2*‐cDNA (NM_013995.2) were constructed by HITRO Bio‐Tech company. The EGFP tag was added to the new initiation codon CDS tail the same as PEGFP‐N1‐*LAMP2*_WT and PEGFP‐N1‐*LAMP2*_Mut. They were used to detect the transient expression of *LAMP2* proteins and to validate the accuracy of predicting new initiation codons. All mutant constructs were sequenced on both strands to verify nucleotide changes.

### 2.7 *LAMP2* transfection and overexpression

Transient overexpression of *LAMP2*_WT, *LAMP2*_Mut, and *LAMP2*_New ATG (new initiation codon position) was achieved in HEK293 (human embryonic kidney 293) cells using Lipofectamine 2000 (Mei5bio: no. MF135) using an empty plasmid as controls. Cells were analyzed 24 h after transfection and transfection efficiency was visualized by immunofluorescence.

### 2.8 Western blotting

Cellular expression of *LAMP2* protein in HEK293 and PBMC (Peripheral blood mononuclear cell) from proband was assessed by WB (Western blotting) and EGFP (Enhanced Green Fluorescent Protein) tag proteins detection to determine whether the predicted initiation codon had initiated translation. Briefly, 30 μg of total protein was extracted from PBMC or transfected HEK293 cells, electrophoresed on SDS‐polyacrylamide gels, and transferred to PVDF (polyvinylidene fluoride) membranes before blocking with dried skimmed milk. Membranes were incubated with anti‐*LAMP2* (CST: no. 49067S, Rabbit Monoclonal Antibody, diluted 1:1000), anti‐GAPDH (CST: no. 5174, Rabbit Monoclonal Antibody, diluted 1:1000), or anti‐EGFP primary antibody (CST: no. 2956, Rabbit Monoclonal Antibody, diluted 1:1000). Membranes were incubated with secondary antibodies carrying HRP (Horseradish Peroxidase) (Signalway antibody, L3012, goat polyclonal antibody, diluted 1:10,000) for 2 h and immunoblots analyzed by ECL (enhanced chemiluminescence) (Thermo: no. 34579).

### 2.9 qPCR

We extracted peripheral blood from the patients while using three healthy females as controls. Total RNA was extracted from PBMC or transfected HEK293 cells by TRIzol (Thermo Fisher: no. 15596018) and reverse‐transcribed to cDNA by M5 Single‐tube qPCR RT kit with gDNA remover (Mei5bio: no. MF011). Reverse transcription primers were used with a separate Oligo 18(dT) (Mei5bio: MF217) to evaluate the processed mRNA. Real‐time quantitative analysis was performed by M5 One‐Step qRT‐PCR Kit (Mei5bio: no. MF049) and relative RNA levels were calculated using 2^−△△CT^. The primer sequence was shown in (Table S1). In addition, cDNA from PBMCs was amplified from the proband and healthy females by 2× M5 HiPer Taq HiFi PCR mix (Mei5bio: no. MF002).

### 2.10 Prediction of cDNA sequence initiation codons

Mutation taster (https://www.mutationtaster.org/) and ATGpr (http://crispr.dbcls.jp/) software were used to predict the position of a new initiation codon following the mutation of the original.

### 2.11 Fluorescence microscopy

Transfection efficiency and EGFP fusion *LAMP2* protein expression were visualized by fluorescence microscopy. The EGFP gene was fused beyond the C‐terminal stop codon of the *LAMP2* plasmid and fluorescence was used to determine *LAMP2* expression and give locational information.

### 2.12 pmCherry‐GFP‐LC3 assay

The pmCherry‐GFP‐LC3 plasmid is acid‐sensitive and dual‐fluorescent. Quenching of GFP fluorescence occurs on the fusion of the lysosome with the autophagosome to form the autolysosome. HEK293 cells were co‐transfected with the *LAMP2*_Mut and pmCherry‐GFP‐LC3 plasmids for 6 h before induction with 200 nM rapamycin for 48 h. Cells were visualized with two‐photon laser confocal microscopy (Zeiss LSM880).

### 2.13 Analysis of species conservation and structural diagram of protein sequence

Conservation analysis of *LAMP2*: p.M1T was performed on the grounds that mutation of the more conserved residues has the greatest likelihood of causing disease. *LAMP2* sequences from a range of species were downloaded from Homologene (https://www.ncbi.nlm.nih.gov/homologene/?term=) and imported into T‐coffee (https://tcoffee.crg.eu/) for alignment. Protein sequence structure maps were plotted via the IBS website (http://ibs.biocuckoo.org/) and domain structure and positions of mutated amino acids are annotated on the figure. Signal peptides were predicted by SignalP (https://services.healthtech.dtu.dk/service.php?SignalP‐5.0) website.

### 2.14 Homology modeling

*LAMP2* (Uniprot: P13473) wild type and *LAMP2*_Mut structures were predicted by ColabFold powered by AlphaFold2 and RoseTTAFold (Mirdita et al., 2022). Protein structures were visualized by Pymol software.

### 2.15 Identification of the X chromosome inactivation pattern

Considering the impact of the X chromosome inactivation pattern on mutant transcripts and clinical phenotypes, we identified the X chromosome inactivation status of the patient. The XCI (X chromosome inactivation) pattern was established by scrutinizing the methylation of CpG dinucleotides within the CAG repeat region of the Androgen Receptor (AR) gene (abbreviation was AR assay) (Elstein et al., 2012). Methylation of various forms is commonly observed on the inactivated X chromosome, particularly on the promoter and CG‐enriched CpG islands. Methylation inhibits transcription of these regions, while the active X chromosome is typically not methylated. Since methylation‐sensitive restriction enzymes HpaII are unable to cut regions containing methylated bases in the methylation restriction site, we analyzed the X chromosome inactivation status based on this differential feature of the active and inactive X chromosomes. Following the digestion of DNA with HpaII and selective amplification of methylated DNA, we amplified the AR gene locus using two distinct primers and scrutinized the resulting PCR products with the ABI 3730xl DNA analyzer and Gene Mapper software. XCI skewing ratio was determined as previously described (Bottillo et al., 2016). XCI percentage of the paternal allele = [(d1/u1)/{(d1/u1) + (d2/u2)}] × 100, which ranges from 0% to 100% (Warburton et al., 2009).

### 2.16 Identification of the ratio of mutations and wild transcripts

The method of identification of the ratio of mutations and wild transcripts was shown in Figure 7b. Briefly, the lymphocytes of the proband (separation method was shown in 2.4) by TRIzol (Thermo Fisher: no. 15596018) and reverse‐transcribed to cDNA (method was shown in 2.9, processed mRNA were evaluated). After amplified the fragment containing the *LAMP2* c.2T>C mutation with cDNA by PCR (*LAMP2*‐pcDNA3.1F:AAGCTTGGTACCGAGCTCGGATCCCCTAGTCTTATGACTCGCACTGAAGCGC;*LAMP2*pcDNA3.1R:TTAAACGGGCCCTCTAGACTCGAGCATAGCGTACTGTGAAATTCATCTGCCA), we inserted the PCR product into pcDNA3.1 to construct the recombinant plasmid (BamHI/XhoI). After transforming the recombinant plasmid into *E. coli*, we randomly selected single colonies and conducted Sanger sequencing using the ABI 3730xl DNA Analyzer. Finally, the ratio of mutations and wild transcripts was analyzed based on the sequencing results.

### 2.17 Statistical analyses

GraphPad Prism9 was used to construct vector plots and statistical analyses were performed by *t* test or one‐way anova. A value of *p* < 0.05 was considered significant.

## 3 RESULTS

### 3.1 Clinical characteristics and examination findings consistent with Danon disease

The somatoscopy examination results showed that the patient had normal muscle strength and no fatigue. The retina and intelligence tests of the patient and her parents were normal (Table 1).

**TABLE 1:** Clinical characteristics and analytical findings of family Pedigree.

		Proband	Proband's father	Proband's mother
Gender		Female	Male	Female
Onset age (years)		18	NA	NA
Age at report (years)		20	40	42
Mutation		c.2T>C; p.M1T	None	None
Extracardiac involvement				
Skeletal muscle (MRC grade)		Normal (5)	Normal (5)	Normal (5)
Intelligence (WAIS‐RC)		Normal (96)	Normal (91)	Normal (94)
Eye		Normal	Normal	Normal
Heart				
ECG		WPW, AVR, VTach	Normal	Normal
Echocardiography				
IVS (mm)		20	6	8
LVPW (mm)		23	7	8
LVEF (%)		49	63	61
LVOT (m/s)		1.8	0.9	1.5
LVEDD (mm)		31	48	53
LVESD (mm)		13	31	34
Analytical findings				
Detection index	Reference ranges			
CK (U/L)	38–240	123	135	116
CKMB (ng/mL)	2.0–7.2	13	<2	<2
AST (U/L)	1–49	46	22	25
ALT (U/L)	1–49	13	22	12
a‐HBDH (U/L)	72–182	429	132	128
LDH (U/L)	124–240	441	162	148
TnI (ng/mL)	0.01–0.023	1.4	0.019	<0.001
NT‐proBNP (pg/mL)	300–450	1100	40	87

Abbreviations: ALT, alanine aminotransferase; AST, aspartate transaminase; AVR, accelerated ventricular rhythm; CK, creatine kinase; CKMB, creatine kinase myocardial band; IVS, interventricular septum; LDH, lactate dehydrogenase; LVEDD, left ventricular end‐diastolic diameter; LVEF, Left Ventricular Ejection Fractions; LVESD, left ventricular end‐systolic diameter; LVOT, Left ventricular outflow tract; LVPW, left ventricular posterior wall; NT‐proBNP, N‐terminal pro‐B type natriuretic peptide; TnI, Troponin I; VTach, ventricular tachycardia; WPW, Wollf–Parkinson–white‐syndrome.

**FIGURE 1:**
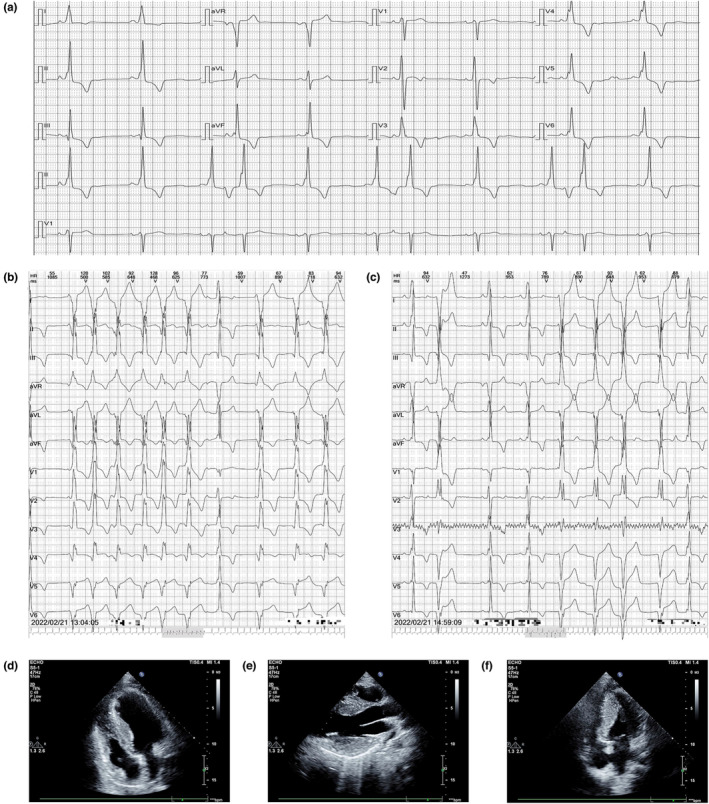
Representative electrocardiogram and echocardiography of proband. (a) An electrocardiogram shows atrial premature contraction, Wolff–Parkinson–White syndrome with an inverted T wave. (b) Accelerated ventricular rhythm. (c) Ventricular tachycardia in dynamic electrocardiography. (d–f) Transthoracic echocardiography shows symmetrical hypertrophy with LVPW of 23 mm, IVS of 20 mm, LVEDD of 31 mm, and LVESD 13 mm.

Electrocardiograph and dynamic electrocardiography showed premature atrial contraction, WPW syndrome with inverted T wave, accelerated ventricular rhythm, and ventricular tachycardia (Figure 1a–c). Echocardiography showed HCM (hypertrophic cardiomyopathy) with left ventricular posterior wall (LVPW) thickness of 23 mm, and a maximum interventricular septal (IVS) thickness of 20 mm (Figure 1d–f). Laboratory tests showed high NT‐proBNP (N‐terminal pro‐B‐type natriuretic peptide, 1100 ng/mL), TnI (cardiac troponin I, 1.4 ng/mL), CK‐MB (creatine kinase myocardial band, 13 ng/mL) and LDH (lactate dehydrogenase, 441 U/L) (Table 1). Other biomarkers of myocardial injury, such as blood glucose and lipids, were within the normal range. The patient had no family history of sudden cardiac death.

**FIGURE 2:**
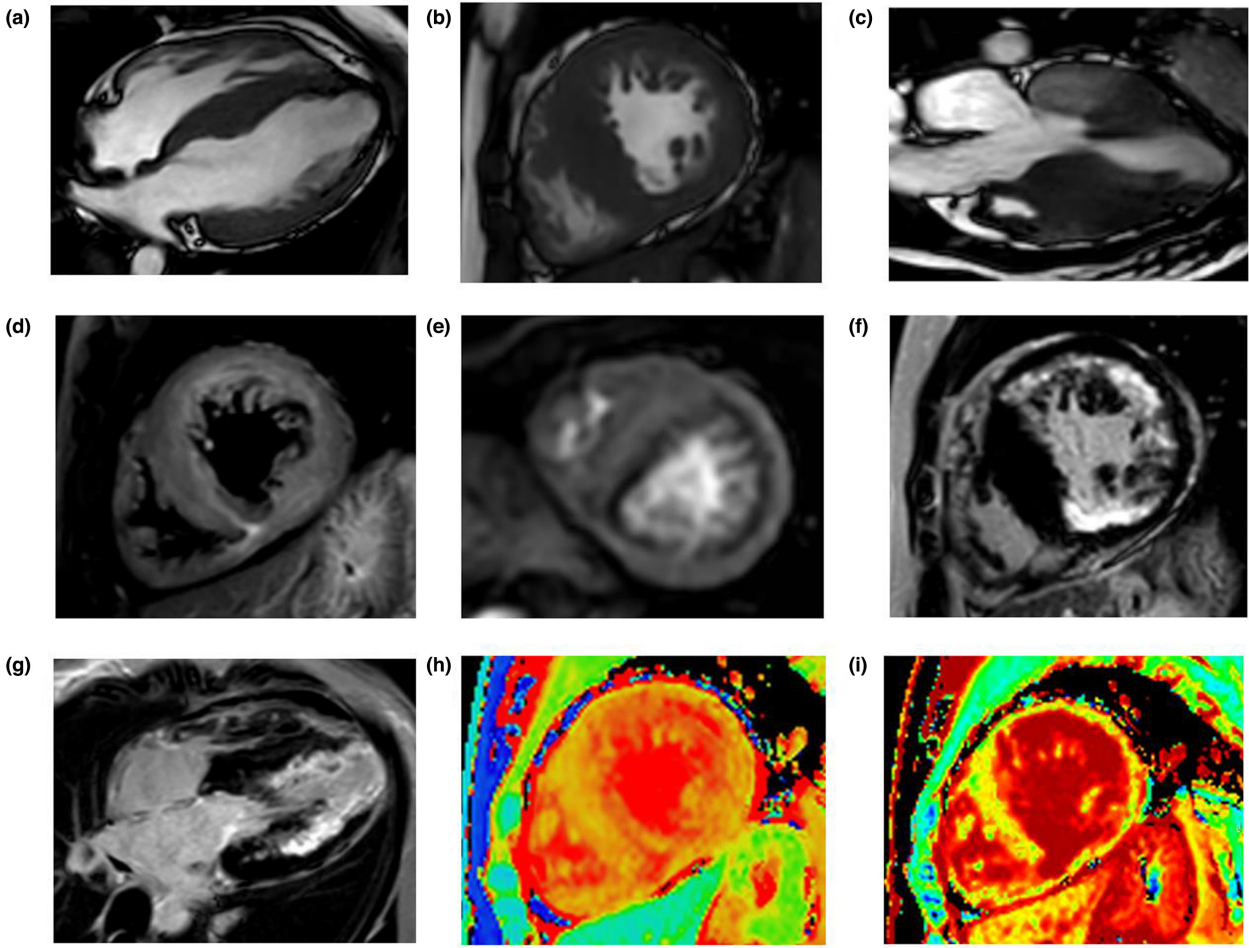
CMR images of the proband. (a–c) Cine images. (d) T2‐weighted image. (e) The first‐pass perfusion image. (f,g) LGE images. (h) T1 map image, and (i) ECV map.

Symmetrical hypertrophic cardiomyopathy was shown by CMR with a maximal thickness of the intraventricular septum of 20 mm at end‐diastole (Figure 2a,b). LV end‐diastolic volume, end‐systolic volumes, and cardiac dimensions were normal, but the LVEF was mildly decreased (49%). No LV outflow tract obstruction could be seen from the 3‐chamber cine image (Figure 2c). An extensive hyper‐T2 signal in the subendocardium‐mid‐wall of LV was seen by the T2‐weighted image (Figure 2d). Significant perfusion defects in the sub‐endocardium, involving almost all segments, could be seen from the first‐pass perfusion image (Figure 2e). LGE (late gadolinium enhancement) was present in the LV free wall but not in the midbasal septum of the subendocardium (Figure 2f) and an increasing LGE tendency from base to apex could be seen from the 4‐chamber LGE image (Figure 2g). LGE volume accounted for 50% of LV myocardial volume. Native T1 (1466 ms) and ECV (47%) values were significantly increased (Figure 2h,i).

Different from the classic hypertrophic cardiomyopathy, the patient has special suggestive findings: (1) electrocardiograph and dynamic electrocardiography showed WPW syndrome; (2) the patient's myocardial enzyme test results indicated elevated CK‐MB, TNI, and LDH but no symptoms of ischemic cardiomyopathy and myocarditis were present; (3) the cardiac MRI results were consistent with the Danon disease characteristics. Further gene sequencing is required to clarify the diagnosis.

### 3.2 A novel pathogenic mutation (*LAMP2* c.2T>C) confirmed Danon disease

**FIGURE 3:**
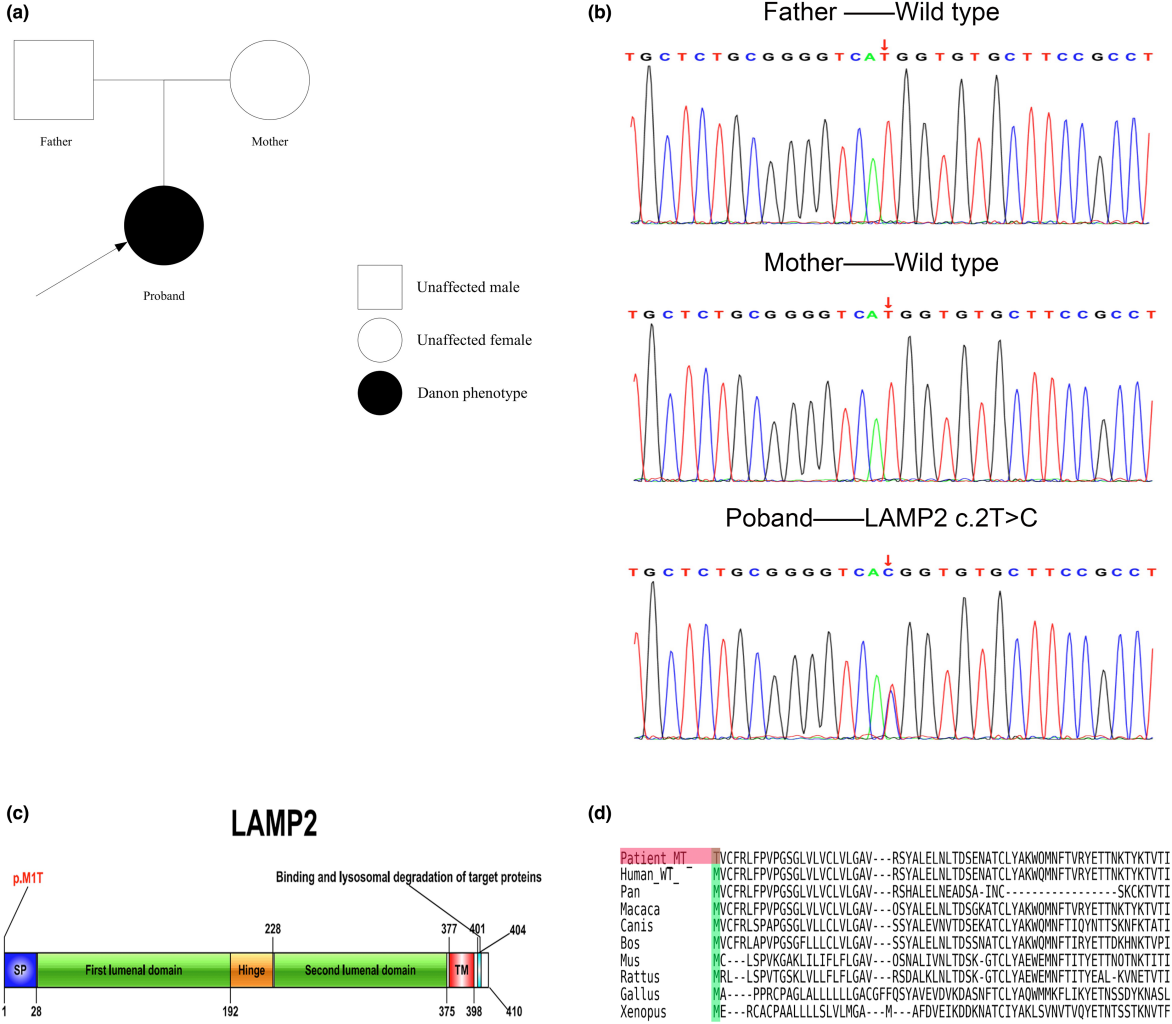
De novo mutations in *LAMP2* were detected in proband. (a) The family tree of the DD pedigree. Square: unaffected male; circle: unaffected female; black filled symbol: affected individual; arrows: proband. (b) Sanger sequencing of family member DNA. Red arrows indicate mutation sites and wild‐type sites. (c) A structure scheme of *LAMP2* protein. SP, signal peptide; p.M1T, threonine replaces methionine; TM, transmembrane domain. (d) Species conservation analysis of the amino acid sequence. Green shaded region: amino acids of each species; Red shaded region: mutated amino acid of the patient.

Whole‐exon sequencing found a de novo mutation of *LAMP2* (*LAMP2* c.2T>C) in the proband. This locus has been associated with DD in OMIM. The mutation was located at the initiation codon and impacted the translation of the entire *LAMP2* transcript. The mutation was novel and has not been reported in any database (dbSNP, 1000 genomes, gnomAD). Neither of the proband's parents carried the mutation nor had they any clinical manifestation of DD (Figure 3a,b, Figure S1), indicating that the mutation had arisen de novo (results of paternity testing for the proband and the parents was shown in Figure S3). The location of the mutation at the initiation codon (protein structure scheme with mutation position shown in Figure 3c) indicates an impact on *LAMP2* localization and expression, in accordance with software predictions. Haploinsufficiency or limited localization of *LAMP2* gives a high probability of association with the disease. Conservation analysis indicated the highly conserved status of the first methionine in the *LAMP2* protein‐coding sequence (Figure 3d), suggesting a likely impact on protein structure and function. ACMG guidelines classify the novel mutation as most likely pathogenic (PVS1−moderate + PS2 + PM2). The above evidence indicates the suitability of the de novo pathogenic mutation to be used as an adjunct for the diagnosis of DD.

According to Matthew RG Taylor's opinion (Taylor & Adler, 2020) the diagnosis of Danon disease is established in a proband with suggestive findings and a heterozygous pathogenic variant in *LAMP2* identified by molecular genetic testing.

### 3.3 *LAMP2*_mutation caused haploinsufficiency in HEK293 and PBMC

*LAMP2* wild‐type and *LAMP2*_mutant plasmids were expressed in HEK 293 cells which have the capacity to express exogenous genes at high levels. Native expression of *LAMP2* mRNA and protein was also measured in PBMCs from patients and healthy controls (age‐matched, three healthy females).

**FIGURE 4:**
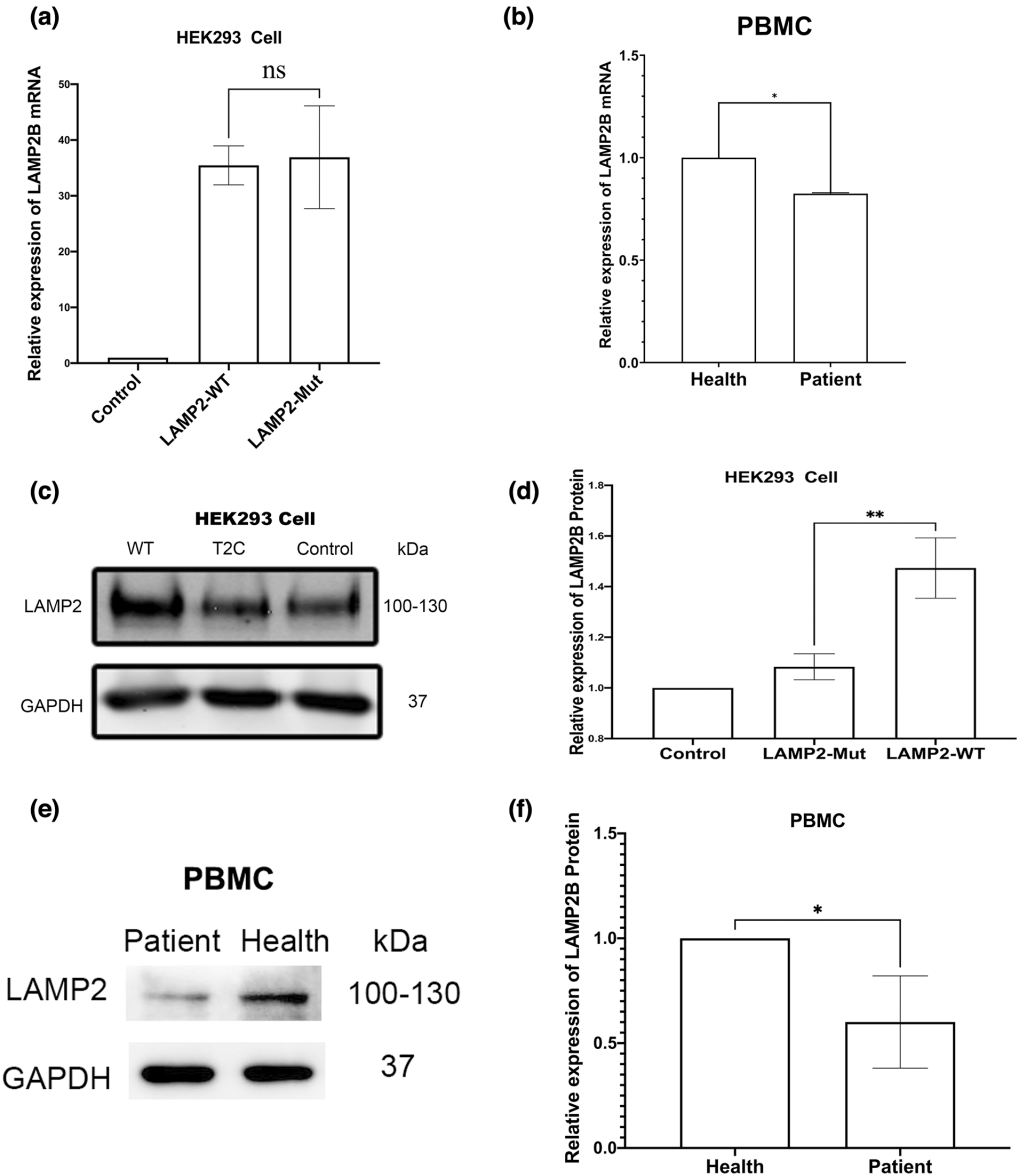
The novel de novo mutation caused *LAMP2* haploinsufficiency in cell experiments. (a) Relative expression of *LAMP2* mRNA in HEK293 cells. (b) Relative expression of *LAMP2* mRNA in PBMCs from the proband and healthy female controls. (c) *LAMP2* protein levels in HEK293 cells after transfection with the *LAMP2* wild type and mutant plasmids from Western blotting results. (d) Histogram of WB results in HEK293 cells. (e) *LAMP2* protein levels in PBMCs from the proband and healthy female controls. (f) Histogram of WB results in PBMC. *GAPDH*, glyceraldehyde‐3‐phosphate dehydrogenase; *LAMP2*, lysosome‐associated membrane glycoprotein 2. *LAMP2*_WT, HEK293 cells transfected with wild‐type *LAMP2* plasmid; *LAMP2*_Mut, HEK293 cells transfected with *LAMP2* mutant plasmid (c.2T>C); PBMC, peripheral blood mononuclear cells; *p < 0.05; **p < 0.01.

The *LAMP2* wild‐type and *LAMP2*_mutant mRNA levels detected by qPCR in HEK293 cells were no different (Figure 4a). However, the patient showed a slight decrease (approximately 20% of the mRNA was degraded) in mRNA levels compared with the healthy female controls (Figure 4b). This difference may be due to in vivo mRNA levels being regulated by X chromosome inactivation but not in vitro.

WB result showed decreased levels of normal functional *LAMP2* protein when the *LAMP2* mutation was present both in HEK293 cells and in PBMCs (Figure 4c–f). This suggests that functional *LAMP2* levels were insufficient. Although the patient's leukocytes cannot replace the cardiomyocytes, the WB results at least in part indicate that the patient developed *LAMP2* haploinsufficiency. The reason for *LAMP2* haploinsufficiency may be: (1) the effect of the mutations on the structure and function of the *LAMP2* protein and (2) the effect of X chromosome inactivation on the *LAMP2* expression level. The mechanisms leading to *LAMP2* haploinsufficiency are worth exploring.

### 3.4 The new initiation codon was first ATG downstream from the original initiation codon, encoding 6 amino acids before terminating translation

Mutations located at the initiation codon usually prevent the protein from translating from the original codon. The key to studying initiation codon mutations is to search for new initiation codons capable of translation.

Two outcomes had been predicted for the new initiation codon: (1) the first ATG downstream from the original position (c. 80–82) predicted by Mutation tasting (Figure S2a) and (2) mutation produces a truncated N terminus but complete C terminus (Figure S2b). EGFP was tagged to the new initiation codon CDS tail to confirm whether the protein translation can be performed. At the same time, the protein carboxy terminus of *LAMP2*_WT and *LAMP2*_Mut was tagged with EGFP as a control. EFGP‐tagged proteins were visualized by fluorescence microscopy imaging and WB.

**FIGURE 5:**
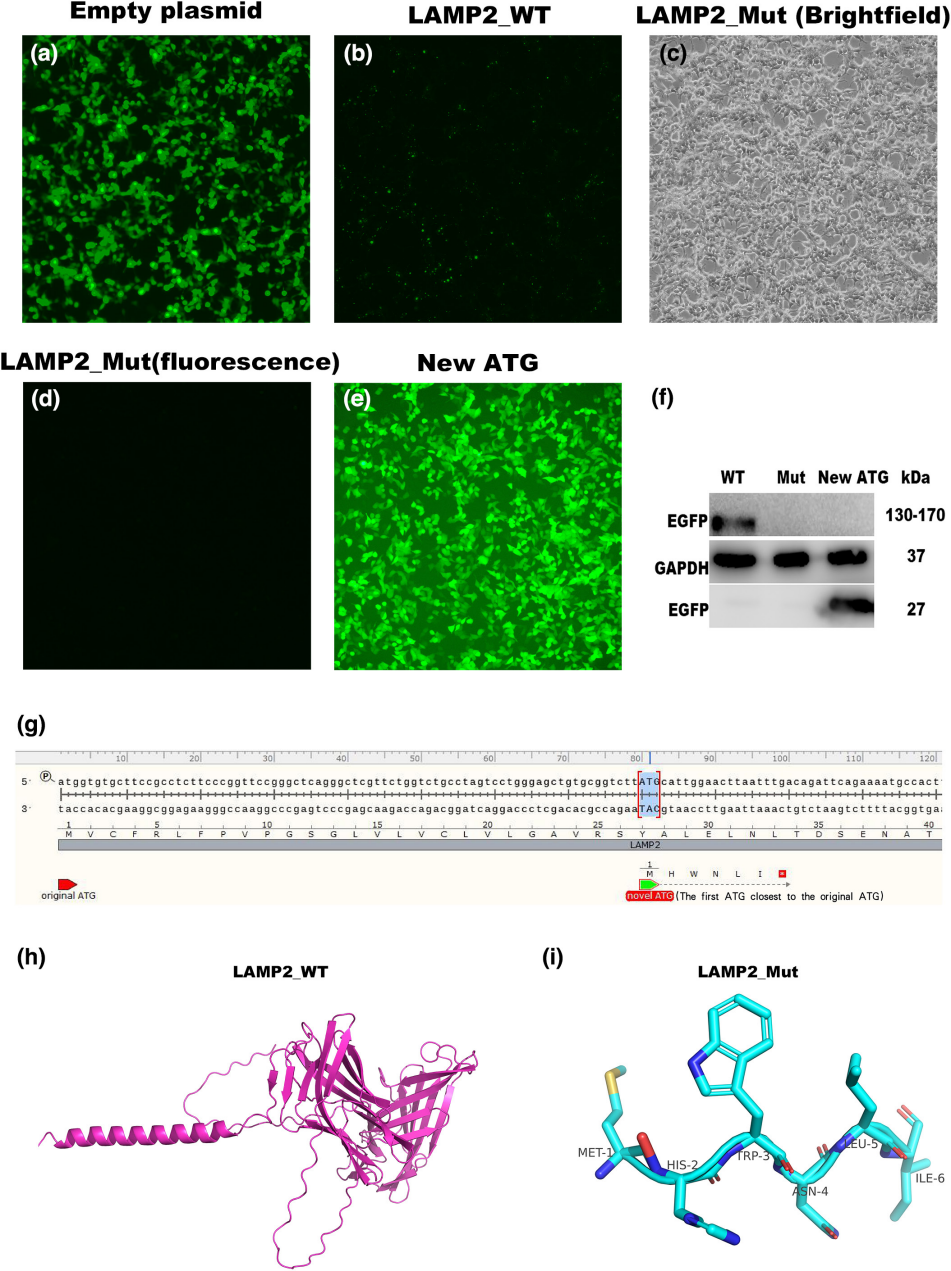
The new initiation codon was first ATG downstream from the original initiation codon which lost almost all the domains of the *LAMP2* protein. (a) Fluorescent level of empty plasmid fused with EGFP. (b) Fluorescent level of *LAMP2*_WT fused with EGFP. (c) Fluorescent level of *LAMP2*_Mut fused with EGFP (bright filed). (d) Fluorescent level of *LAMP2*_Mut fused with EGFP (fluorescence filed). (e) Fluorescent level of *LAMP2*_new ATG fused with EGFP. (f) The protein level of EGFP in HEK293 cells after transfected with the *LAMP2* plasmid (WT, Mut, and New ATG) were evaluated using Western blot analysis. (g) Schematic diagram of the new initiation codon position. (h) The 3D structure of the *LAMP2*_WT protein is shown in cartoon mode. (i) The 3D structure of the *LAMP2*_Mut protein is shown in cartoon mode. Mut, mutant type; WT, wild type; New ATG represents new initiation codon.

*LAMP2*_Mut, whether bright field and fluorescence, did not produce a greater level of green fluorescence than the empty plasmid or the *LAMP2*_WT (Figure 5a–d). Thus, the *LAMP2*_Mut plasmid could not translate until the carboxy terminus, indicating the presence of a premature termination codon. WB results confirmed this conclusion and no EGFP protein was detected in the *LAMP2*_Mut group (Figure 5f). By contrast, the detection of green fluorescence showed that the first ATG downstream from the original initiation codon produced an EGFP fusion protein (Figure 5 e,f). To facilitate the understanding, we made a schematic diagram of the new initiation codon position (Figure 5g). We predicted the *LAMP2*_Mut by alphafold2 software. In contrast to the wild type, its structure almost completely missing all domains, almost impossible to exert its original function (Figure 5h,i).

Our results show that the mutations cause truncation of *LAMP2* protein and could not translate normal proteins, which are estimated to be degraded quickly.

### 3.5 Mutant proteins (*LAMP2*_Mut) failed to perform normal *LAMP2* protein function, impairing the late autophagic process

**FIGURE 6:**
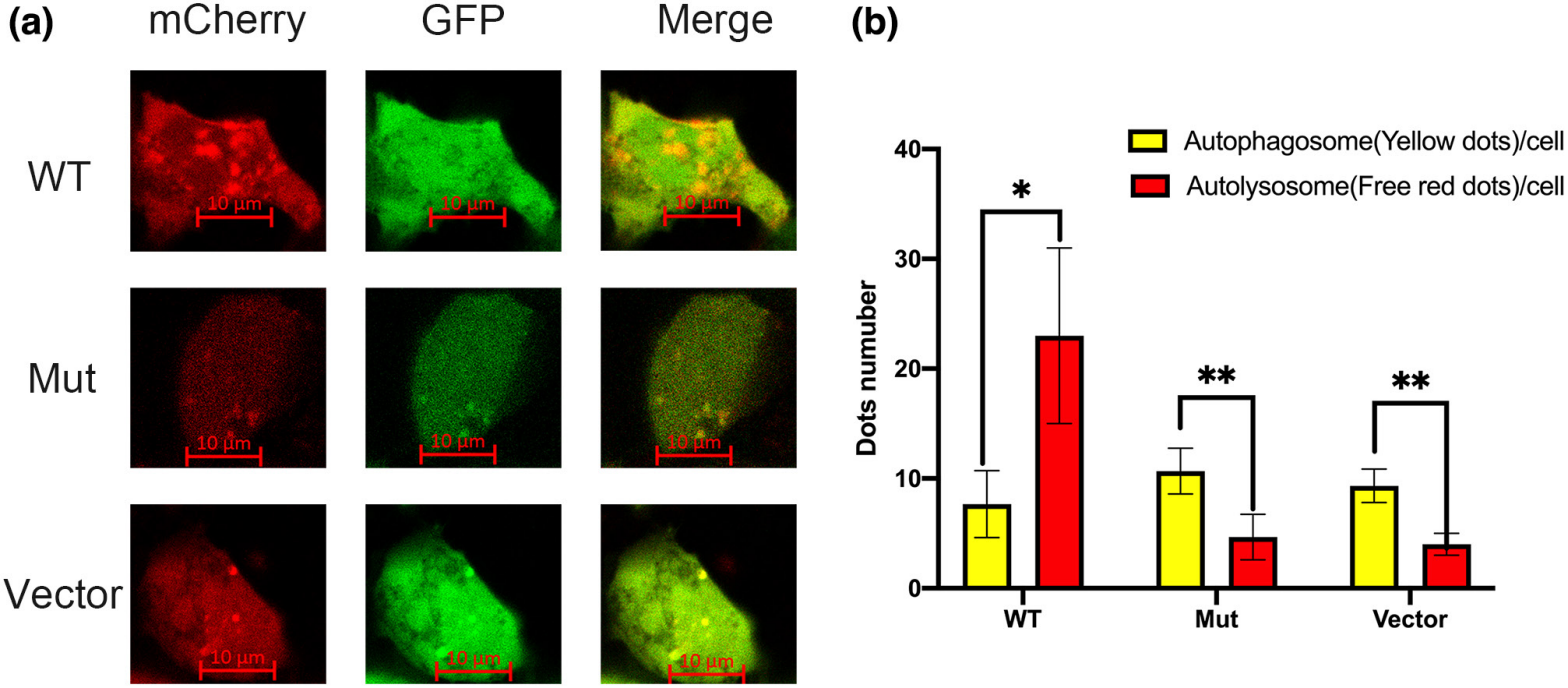
Result of autophagy dual fluorescent between *LAMP2*_WT, *LAMP2*_Mut, and vector. (a) HEK293 cells were cotransfected with mCherry‐GFP‐LC3B and empty, *LAMP2*_WT or *LAMP2*_Mut plasmids for 6 h before induction by 200 nM rapamycin for 48 h. The formation of autophagosomes (yellow fluorescence) and autolysosomes (red fluorescence) was detected by laser confocal microscopy. (b) Quantification curve of the autophagosomes (yellow fluorescence) and autolysosomes (red fluorescence) dots. Values are presented as the mean with SD. Autolysosomes (red fluorescence) dots compared with autophagosomes (yellow fluorescence) dots, *p < 0.05, **p < 0.01. Mut, mutant type; WT, wild type.

The *LAMP2* and mCherry‐GFP‐LC3B autophagy dual‐fluorescent plasmids were co‐transfected into HEK293 cells and autophagic flow was observed by Laser confocal microscopy. Fluorescence from GFP is quenched in an acidic environment but that from mCherry is not. Thus, red fluorescence in late autophagy represents autolysosome number and yellow fluorescence represents autophagosome number. If the late autophagic flow is blocked, increased yellow fluorescence is observed. Laser confocal results showed increased yellow fluorescence in *LAMP2*_Mut compared with *LAMP2* WT (Figure 6), suggesting impairing late‐stage autophagic flow. Thus, *LAMP2*_Mut could not perform normal *LAMP2* protein function which regulates the formation of autolysosome.

### 3.6 28% of mutant X chromosomes remained active

Previous studies have confirmed that missense mutations (*LAMP2* c.2T>C) located at the initiation codon are considered null mutations based on the missing *LAMP2* structure and function results.

**FIGURE 7:**
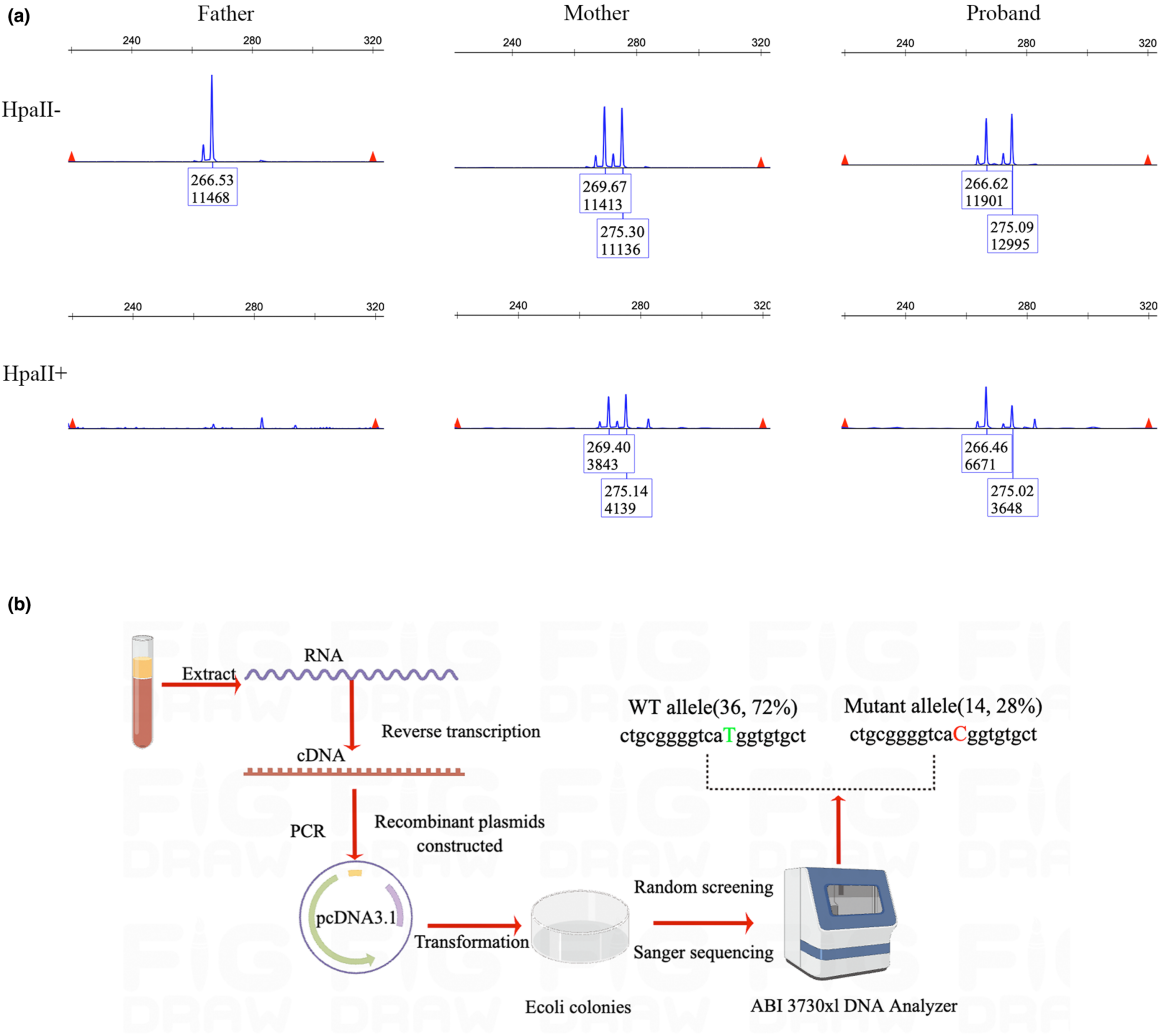
Identification of X chromosome inactivation pattern and active proportion of the mutant X chromosome. (a) XCI pattern was detected by AR assay, and 266 bp peak for AR was observed by examining the undigested PCR product of the father, but no peak for the HpaII digested product. The undigested PCR product of the mother has two peaks of 269 and 275 bp, and two decreased peaks of 269 and 275 bp were observed with the HpaII digested product. The undigested PCR product of the proband gave two peaks of 266 and 275 bp. One X‐chromosome linked with the 266 bp peak of AR was inherited from the father and 275 bp from the mother. Decreased peaks of 266 and 275 bp were observed in the digested product of proband. XCI percentage of the paternal allele = [(d1/u1)/{(d1/u1) + (d2/u2)}] × 100, The inactivation of the paternal X chromosome was found to be 66%, while that of the maternal X chromosome was 34%. For each box, the top represents the PCR product length, and the bottom represents the peak area. (b) Illustration representation of the method used to identify the ratio of mutated and wild‐type transcripts (the drawing is done by Figdraw). The reverse transcription primer was using Oligo (dT), and 50 positive single clones were randomly selected. The results indicate that 28% of transcripts are mutant and 72% wild type. RNA: Here it refers to the mRNA. HpaII−, results without digestion by HpaII, HpaII+, results with digestion byHpaII.

The extent of X chromosome inactivation is a critical factor that determines whether mutations and wild‐type alleles are equally assigned and expressed. This can be a primary cause of phenotypic differences observed among female carriers. We identified the X chromosome inactivation state in the proband using an AR assay, which allowed us to determine the extent of inactivation of the X chromosome from both paternal and maternal lines. The inactivation rate of the X chromosome paternal inherited was 66% (maternally inherited was 34%) in the blood leukocytes of our patient. Analysis of the proband's inactivated X chromosome revealed no skewing (Figure 7a). We then obtained cDNA by reverse transcribing mRNA from patient leukocytes. After constructing recombinant plasmids, we randomly selected 50 positive monoclonal colonies for Sanger sequencing. The results showed that 28% (14/50) of mutant transcripts remained active in the patient's leukocytes (Figure 7b). Given the approximately 80% reduction in mRNA observed in the patient, approximately 57.5% of the wild‐type transcripts remained active in comparison to healthy women. This was consistent with the results of the WB.

## 4 DISCUSSION

In the present study, we investigated a young female patient diagnosed with Danon disease, elucidation the identification and functional analysis of the mutations. Similar to the findings of a nationwide survey on Danon disease in Japan, the most typical clinical presentation of the proband was cardiac involvement, especially hypertrophic cardiomyopathy (Sugie et al., 2018). Myopathy and mental retardation were not found in this case, which is also in line with the reported results of a relatively low probability (9%) of both clinical manifestations in female patients (Sugie et al., 2018).

There is no doubt that myocardial tissue obtained from the endocardial biopsy is the best sample for the diagnosis of DD. However, due to patient reluctance, particularly among young females, regarding the acceptance of invasive examination methods, peripheral blood leukocytes may serve as a viable alternative. Nevertheless, it is important to acknowledge the potential variability across tissues. There is debate about whether peripheral leukocytes can serve as an alternative diagnosis of the myocardium. But some researchers have shown that *LAMP2* expression testing by WB in peripheral WBCs may be used as evidence of endogenous insufficiency if the patient is unwilling to undergo an endocardial biopsy (Gurka et al., 2020; Regelsberger et al., 2009; Xu et al., 2019).

The patient had a de novo mutation (*LAMP2*: c.2T>C) which was one of the genetic features of Danon disease. The identified mutation leads to a substitution of methionine with threonine (p.M1T) at the initiation codon. Additionally, this alteration may impact the normal initiation of translation, indicating that it could be a null mutation. Initiation codon mutations may interfere with the initiation of protein translation, cause N‐terminal truncation or displace the mRNA reading frame (Richards et al., 2015). In the current case, mutation of the first ATG initiation codon triggered the re‐selection and translational initiation at other initiation codons within the coding sequence (CDS). Although the translation was restarted, only six amino acids were translated. The dual‐fluorescence autophagy indicator system, mCherry‐GFP‐LC3, confirmed that *LAMP2*‐Mut failed to function as a wild‐type *LAMP2* protein in regulating autophagy. These pieces of evidence suggest that the missense mutation may be null.

We hypothesize that the observed decrease in *LAMP2* expression in the patient may result from *LAMP2* haploinsufficiency due to a heterozygous null mutation. Our conclusions are consistent with the findings of Kazuma Sugie (Sugie et al., 2016). Our patient carried a missense mutation located at the initiation codon, and the mutated transcript failed to translate the normally functional *LAMP2* protein. In addition, we also examined the extent of X chromosome inactivation in the patients. While the results suggest that the skewed X chromosome inactivation was not reached, we found that the paternal X chromosome was inactivated by 66%, which is consistent with the fact that only 28% of the mutant transcripts were detected. Despite the protective effect of mutational X chromosome inactivation, only 66% of the normal *LAMP2* protein was produced, which was insufficient to meet the metabolic requirements of the patient's cardiomyocytes. Deficiency of the *LAMP2* protein gradually aggravates the myocardial injury phenotype by increasing in proportion to age (Sugie et al., 2018). We speculate that in the early stages of the disease, cells can relieve the arrest of autophagic flow through compensatory mechanisms. However, the gradual accumulation of autophagic vacuoles eventually leads to cardiomyocyte hypertrophy, apoptosis, and the development of cardiomyopathy. Heterozygous mutations causing defects or null function in the protein of *LAMP2* were the genetic basis of Danon disease, haploinsufficiency, and X chromosome inactivation skewing determine the severity of the patient's phenotype (Sugie et al., 2016).

Heterozygous females with mutations in the X‐linked allele are typically mildly affected or remain unaffected due to compensation from the wild‐type allele (Hedberg Oldfors et al., 2015). As a result, females usually have a later onset than males. The mean age at presentation of Danon disease was 38 ± 12 years in females compared with 17 ± 7 years in males (Sugie et al., 2002). Our female patient had an early onset (18 years) than most female patients. The pathogenesis of early onset cardiomyopathy in women with Danon disease is still unclear, but based on the existing insights, it is likely to be caused by variable genotype–phenotype relation of different mutations, different X chromosome inactivation (XCI) patterns (Fu et al., 2016), and detrimental inhomogeneous distribution of *LAMP2* deficiency (Hedberg Oldfors et al., 2015). For our patient, the de novo mutation was a missense mutation located in the initiation codon that affected the translation initiation process and was confirmed to be a null mutation. Although current insight indicated that nonsense, frameshift, and large deletion/duplication mutations were associated with an early onset of Danon disease, whereas splicing and missense mutations showed a trend of a later disease onset (D'Souza et al., 2014), missense mutations located at the start codon confirmed as null mutations are associated with an early onset in our case. For X chromosome‐linked genetic disorders, highly skewed XCI (90% or higher) is increased in women with abnormal X chromosomes in a manner that preserves the normal X chromosome (or autosomal) dosage (Brown et al., 2001; Sun et al., 2022; Warburton et al., 2009; Xu et al., 2019). Skewed XCI in female patients associated with normal *LAMP2* expression and normal clinical phenotype has been reported (Majer et al., 2012; Xu et al., 2019). The wide spectrum of clinical manifestations in heterozygous women depends on the pattern of X chromosome inactivation (Fu et al., 2016). Our patient approached but did not reach the skewed X chromosome inactivation, 28% of the mutant transcripts were expressed. *LAMP2* haploinsufficiency results from an active and null mutational X chromosome may be responsible for the early onset in this patient. This conclusion is similar to Luis Fernandez's report about early onset women with Danon disease (Fernandez et al., 2021).

In conclusion, we described a de novo *LAMP2* mutation in a female with Danon disease. The diagnosis of Danon disease is based on the identification of *LAMP2* gene mutations by NGS sequencing and special suggestive findings. The missense mutation in the initiation codon was confirmed to be null and unable to encode the normal *LAMP2* protein through in vitro functional assay. Using the AR assay and sequencing, we verified that the X chromosome with the *LAMP2* mutation was approached but did not reach skewing inactivation and only 28% of the mutant transcripts were expressed. The patient's early onset hypertrophic cardiomyopathy was likely determined by the haploinsufficiency of *LAMP2* and the XCI inactivation pattern. Identifying and functional analysis of *LAMP2* mutations in female Danon disease can broaden our understanding of this rare disease.

## 5 STUDY LIMITATIONS

The current study has some limitations. IPSCs reprogramming from patient skin fibroblasts followed by differentiation into cardiomyocytes would be a superior expression system to HEK293 cells and PBMCs. As the patient was young and unwilling to undergo myocardial biopsy, we were not able to obtain cardiac tissue, instead with leukocytes for a surrogate study. It is worth mentioning that the XCI patterns and *LAMP2* expression levels may be different in cardiomyocytes and leukocytes. Moreover, the generation of experimental animal models with targeted mutations that recapitulate the patient's clinical phenotype would allow clearer observation of the pathological progression.

## 6 CONCLUSION

In conclusion, the current study identified a novel de novo mutation of *LAMP2* in Danon disease. The functional analysis confirmed the haploinsufficiency of *LAMP2*. We found a new initiation codon for translation was the first ATG downstream of the original. Unfortunately, the new start codon encodes only six amino acids and fails to form functional polypeptides and proteins to fulfill the cellular autophagic process. Although the inactivation X chromosome carrying the mutation was not significantly skewed, it downregulated the expression of the mutant transcript. X chromosome inactivation and null mutations were responsible for early onset in this female Danon patient.

## AUTHOR CONTRIBUTIONS

**Yongxiang Wang**: drawing figures and writing the manuscript, completing the main experimental work. **Yu Peng**: case collection and description. **Zixian Chen**: CMR Imaging and Analysis. **Zhiyu He**: Echocardiographic imaging and analysis. **Jin Xu**: WB and qPCR work. **Youqi Zhu**: PBMC extract. **Dongdong Yan**: Blood collection. **Runqing Wang**: WB and qPCR work. **Piyi Zhang**: Laser‐based confocal imaging analysis, Androgen Receptor Assay, determining the proportion of wild and mutant transcripts. **Ming Bai**: Project design and financial support. **Zheng Zhang**: Project design and financial support.

## FUNDING INFORMATION

This work was supported by the Clinical Cooperative Pilot Project of Traditional Chinese and Western Medicine for Major Diseases (no. Administration of State Administration of Traditional Chinese Medicine [2018], no. 3), National Key R&D Program of China (no. 2018YFC1311505), Gansu Provincial Clinical Research Center for Cardiovascular Diseases (no. 18JR2FA005), GanSu Province Health Industry Scientific Research Plan Project (no. GSWSKY‐2019‐15), The First Hospital of LanZhou University (no. ldyyyn2020‐28), Department of Science and Technology of GanSu Province (no. 20JR5RA357).

## CONFLICT OF INTEREST STATEMENT

The authors declare that they have no known competing financial interests or personal relationships.

## Supporting information


Table S1.

Figure S1.

Figure S2.

Figure S3.

## Data Availability

The raw data that support the conclusions of this article are available from the authors, without undue reservation.
