# Supplementary material for: Identification and functional analysis of a novel de novo missense mutation located in the initiation codon of LAMP2 associated with early onset female Danon disease
Source: Mol Genet Genomic Med. 2023 Jun 8;11(9):e2216. doi: 10.1002/mgg3.2216 (PMC10496070; doi:10.1002/mgg3.2216)
Supplement: Supplementary file 1 — Table S1. [file MGG3-11-e2216-s002.docx]

| The primer name | Sequence (5’-3’) |
| --- | --- |
| *LAMP2*-QF | ATTTGGTTAATGGCTCCG |
| *LAMP2*-QR | TGGGCTGTAGAATACTTTCC |
| *GAPDH*-F | CAAGGGCATCCTGGGCTACACT |
| *GAPDH*-R | CTCTCTCTTCCTCTTGTGCTCTTGC |
| *β-actin*-F | CCTGGCACCCAGCACAAT |
| *β-actin*-R | GGGCCGGACTCGTCATAC |

**Supplementary Table 1**
