# Supplementary figures and images for: Identification and functional analysis of a novel de novo missense mutation located in the initiation codon of LAMP2 associated with early onset female Danon disease
Source: Mol Genet Genomic Med. 2023 Jun 8;11(9):e2216. doi: 10.1002/mgg3.2216 (PMC10496070; doi:10.1002/mgg3.2216)

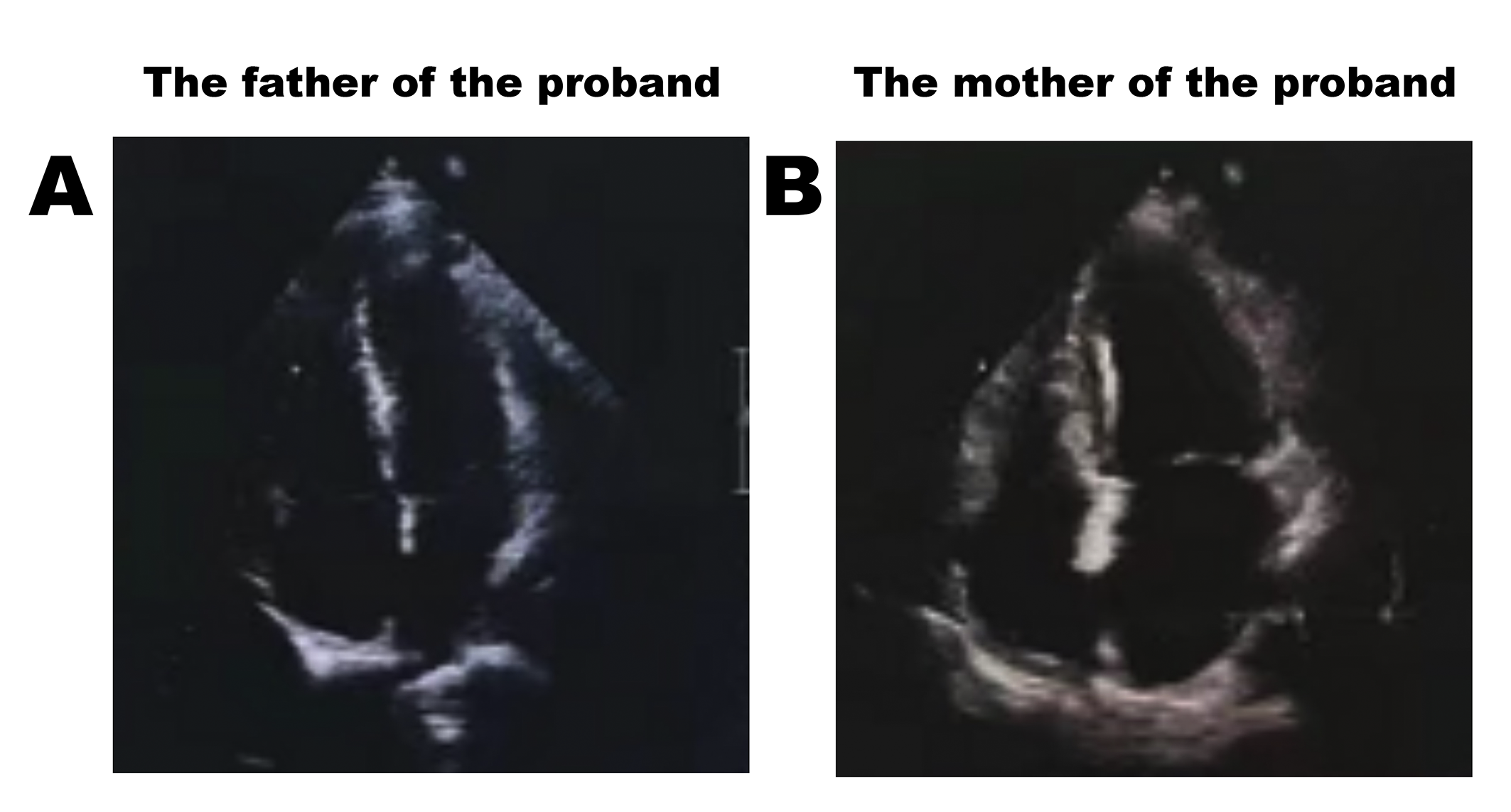

Supplement: Supplementary file 2 — Figure S1. [file MGG3-11-e2216-s003.tif]

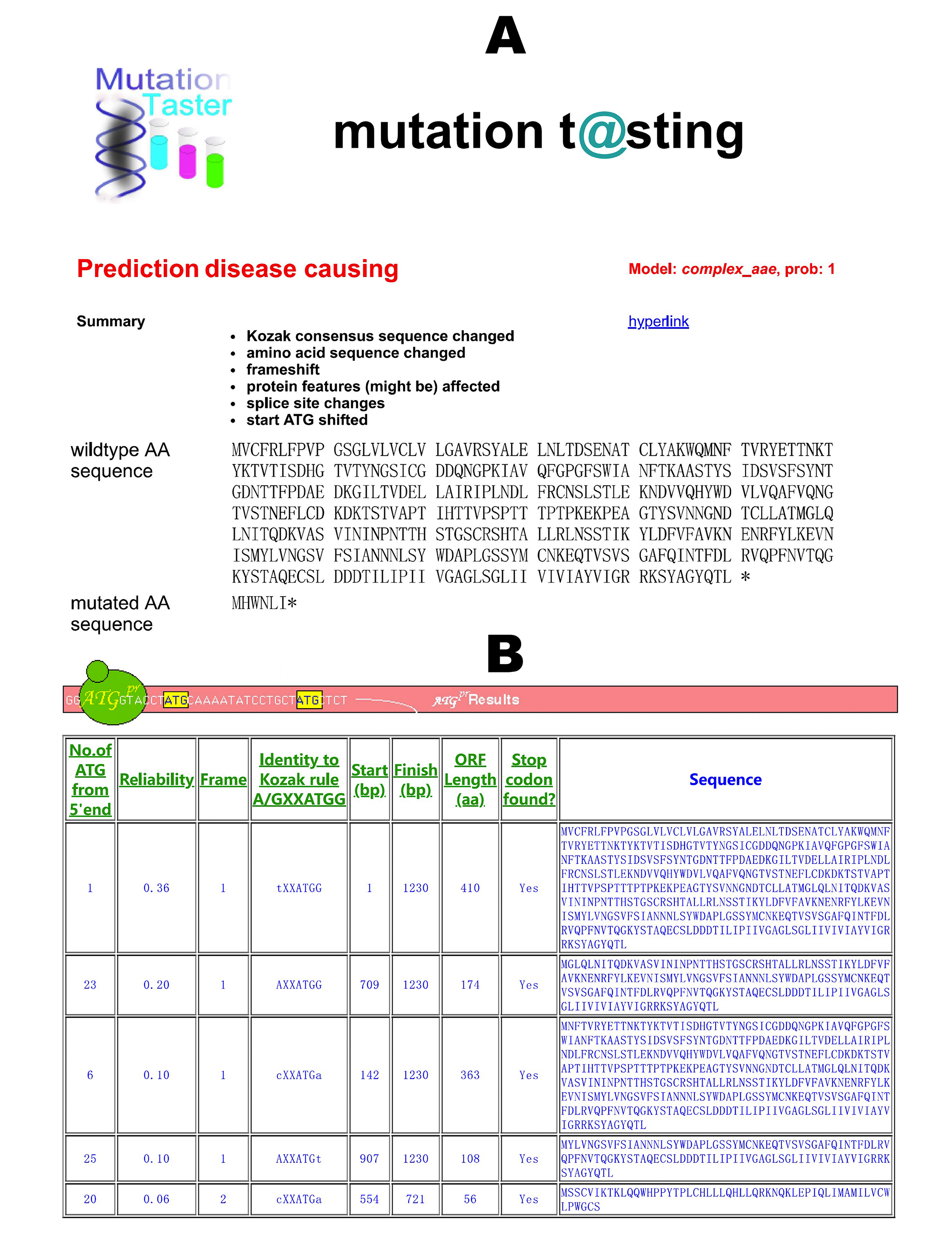

Supplement: Supplementary file 3 — Figure S2. [file MGG3-11-e2216-s001.tif]

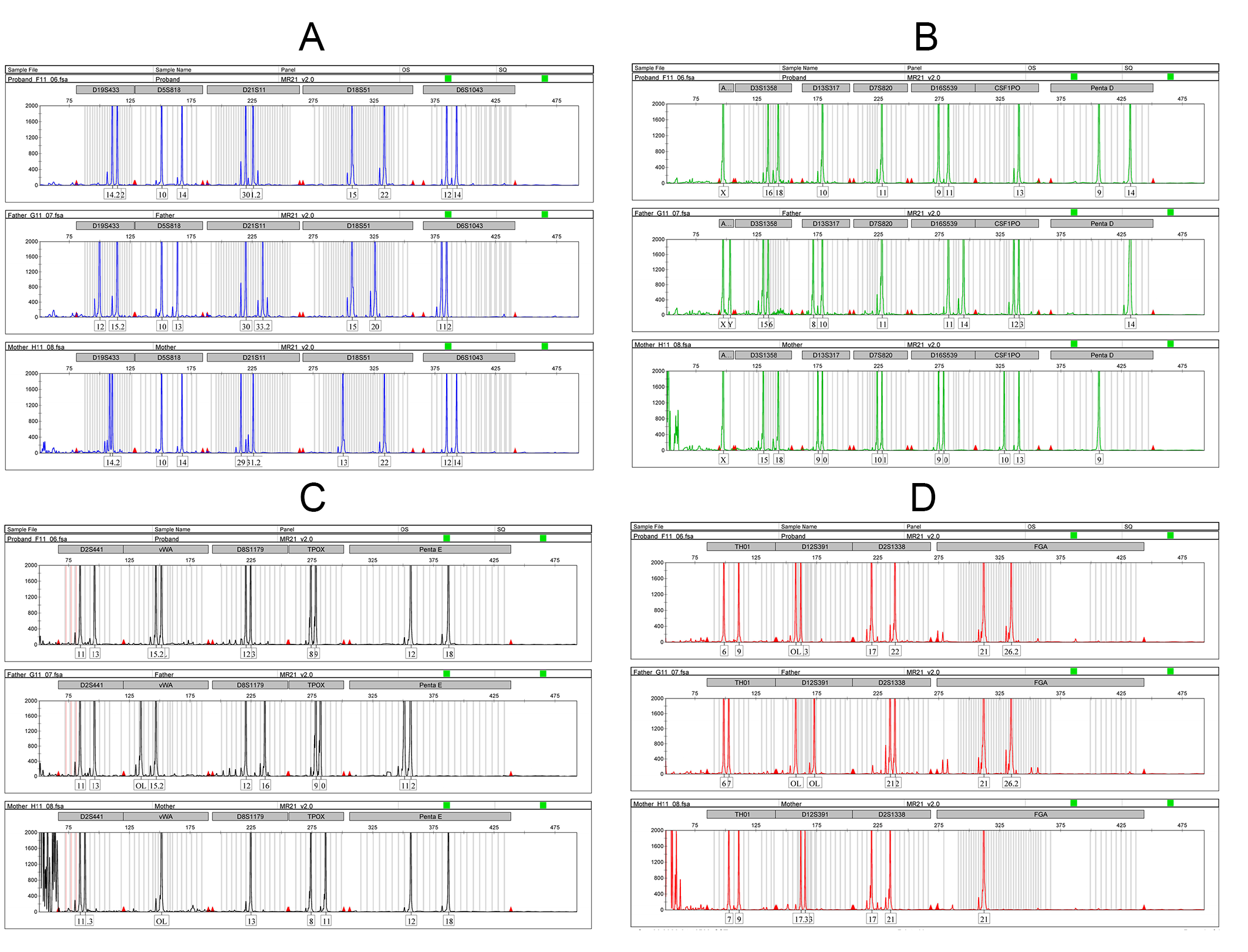

Supplement: Supplementary file 4 — Figure S3. [file MGG3-11-e2216-s004.tif]
